# The Potential Role of Advanced Glycation End Products in the Development of Kidney Disease

**DOI:** 10.3390/nu17050758

**Published:** 2025-02-21

**Authors:** Yibin Ma, Xinyu Wang, Shan Lin, Lei King, Liegang Liu

**Affiliations:** 1 Department of Nutrition and Food Hygiene, Hubei Key Laboratory of Food Nutrition and Safety, School of Public Health, Tongji Medical College, Huazhong University of Science and Technology, Wuhan 430030, China; myb@hust.edu.cn (Y.M.); xywang_@hust.edu.cn (X.W.); d202081512@hust.edu.cn (S.L.); d202281802@hust.edu.cn (L.K.); 2 Ministry of Education Key Laboratory of Environment and Health, School of Public Health, Tongji Medical College, Huazhong University of Science and Technology, Wuhan 430030, China

**Keywords:** advanced glycation end products (AGEs), kidney function, kidney disease

## Abstract

Advanced glycation end products (AGEs) represent a class of toxic and irreversible compounds formed through non-enzymatic reactions between proteins or lipids and carbonyl compounds. AGEs can arise endogenously under normal metabolic conditions and in pathological states such as diabetes, kidney disease, and inflammatory disorders. Additionally, they can be obtained exogenously through dietary intake, particularly from foods high in fat or sugar, as well as grilled and processed items. AGEs accumulate in various organs and have been increasingly recognized as significant contributors to the progression of numerous diseases, particularly kidney disease. As the kidney plays a crucial role in AGE metabolism and excretion, it is highly susceptible to AGE-induced damage. In this review, we provide a comprehensive discussion on the role of AGEs in the onset and progression of various kidney diseases, including diabetic nephropathy, chronic kidney disease, and acute kidney injury. We explore the potential biological mechanisms involved, such as AGE accumulation, the AGEs-RAGE axis, oxidative stress, inflammation, gut microbiota dysbiosis, and AGE-induced DNA damage. Furthermore, we discuss recent findings on the metabolic characteristics of AGEs in vivo and their pathogenic impact on renal function. Additionally, we examine the clinical significance of AGEs in the early diagnosis, treatment, and prognosis of kidney diseases, highlighting their potential as biomarkers and therapeutic targets. By integrating recent advancements in AGE research, this review aims to provide new insights and strategies for mitigating AGE-related renal damage and improving kidney disease management.

## 1. Introduction

Advanced glycation end products (AGEs) are toxic and irreversible compounds formed through non-enzymatic reactions, known as the Maillard reaction, between proteins or lipids and carbonyl compounds such as glucose, fructose, and glyoxa [1,2]. AGEs can form endogenously through normal metabolism or due to abnormal conditions, such as diabetes, kidney disease, and other inflammatory diseases [1,2,3]. They can also enter the body through certain foods, such as barbecue, cheese, and those high in fat and sugar [1,2,3]. Modern dietary habits lead to the increase and accumulation of exogenous AGEs. Research indicates that the buildup of AGEs in the body is closely linked to various common chronic non-communicable diseases, including cardiovascular diseases (CVDs) [4], chronic kidney diseases (CKDs) [5], neurodegenerative diseases [6], cancer [7], and diabetes and its complications [3,8]. AGEs are involved in several metabolic processes within the body. The kidneys are primarily responsible for the metabolism and excretion of AGEs and are also one of the organs most vulnerable to their effects [2]. Research has shown a significant connection between AGE levels and kidney dysfunction, which may be a key factor contributing to the development of kidney disease [6,9,10]. However, most studies have focused primarily on the association of AGEs and the risk factors of kidney disease, such as diabetes and hypertension, with limited research into their effects on kidney function. This review will provide new information on the impact of AGEs on kidney function, including their role in diabetic nephropathy (DN), CKD, acute kidney injury (AKI), and other kidney diseases, as well as the possible mechanism of AGE-induced renal damage, providing new insights and strategies for the early diagnosis, treatment, and prognosis of kidney diseases.

## 2. Methods

We conducted comprehensive searches in databases, including Pubmed and the Web of Science, to retrieve articles relevant to AGEs and kidney diseases. The search period spanned from the earliest available records up to December 2024. The following keywords were employed: “Advanced glycation end products”, “Kidney function”, “Renal function”, “Kidney disease”, and “Renal disease”. At the same time, according to the selected studies before and after citation retrieval, we delved as far as possible into the relevant research.

## 3. Sources, Absorption, Metabolism, and Structure of AGEs

### 3.1. Sources of AGEs

Currently, over 40 distinct AGEs have been discovered, including Carboxyethyl lysine (CEL), carboxymethyl lysine (CML), and pentosidine [1]. They can be categorized into two types: exogenous and endogenous. Exogenous AGEs are acquired from external sources that the body absorbs, while endogenous AGEs are produced internally through processes such as the Maillard reaction or other pathways involving reducing sugars and proteins, lipids, or nucleic acids under normal physiological conditions [1,11,12,13]. Factors such as aging, inflammation, renal failure, oxidative stress, high-fat diets, processed foods, excessive alcohol consumption, and smoking can increase the endogenous or exogenous production of AGEs [14,15].

Abundant evidence indicates that dietary-derived AGEs contribute to the human AGE pool [1,12]. Low-moisture thermal processing approaches, such as frying, grilling, and baking, can substantially increase the AGE content in food [16,17,18]. In addition to the cooking methods, the food ingredients themselves are also crucial factors in determining the AGE content. Almost all foods contain AGEs. Animal-based foods that are high in fat and protein typically contain abundant AGEs [19]. For example, meat and milk contain over 200.0 mg/kg protein, and they tend to form new AGEs during the cooking process [19] In contrast, even after cooking, vegetables and fruits have relatively low AGE contents (of below 100.0 mg/kg protein) [19]. Additionally, ultra-processed food has the highest CML content (up to over 1000.0 mg/kg protein) [18]. The content of AGEs in food is significantly higher than that formed in the body [12].

### 3.2. Absorption and Metabolism of AGEs

The absorption and reaction mechanisms of AGEs within the intestine are highly intricate. Notably, diverse types of AGEs exhibit varying patterns of absorption.

Food-borne AGEs with a molecular weight of below 5 kDa are absorbed in their free form in the gastrointestinal tract, while those exceeding this threshold form polymer complexes by binding to proteins [12,20]. AGEs in their bound state undergo hydrolysis within intestinal epithelial cells (IECs), facilitated by various peptidases.

Furthermore, these bound AGEs can cross the IEC barrier and enter the bloodstream by endocytosis, the paracellular route, or diffusion [12,20]. Free AGEs, with their relatively low affinity for lysine transporters, can be rapidly absorbed by simple diffusion [12,21]. Both the AGEs bound to proteins and those bound to peptides are enzymatically hydrolyzed into smaller fragments before being absorbed by IECs [12,21].

Subsequently, AGEs, in combination with the absorbable portion, may penetrate the circulatory system, thereby posing a potential threat to human health [22,23]. The unabsorbed fraction of the AGEs can be partly metabolized by the intestinal flora and subsequently eliminated from the body via fecal excretion [24]. Around 10% of AGEs are taken up by the small intestine and then make their way into the bloodstream. About one-third of the products are excreted from the body through urine, while the remaining two-thirds stay in the body [25]. Once in circulation, AGEs tend to accumulate in the kidney and liver. The human body primarily metabolizes AGEs through cellular clearance and renal excretion. However, in patients with nephropathy, this excretion is significantly impaired [26]. Research indicates that the glomerulus continuously filters free CML and pentose, while lysosomes are involved in degrading both free and peptide-bound AGEs, allowing dietary AGEs to be reabsorbed in the proximal renal tubules [27]. It is calculated that, in healthy individuals, the amount of AGEs excreted through the kidneys accounts for approximately 30% of the total intake, while in patients with kidney impairment, the amount of AGEs excreted is only 5% [25].

### 3.3. Structure of AGEs

AGEs are a diverse class of molecules formed through a non-enzymatic glycation reaction between reducing sugars and the amino groups of proteins, lipids, or nucleic acids [14]. This process, known as the Maillard reaction, results in the formation of a wide range of chemical structures that differ in complexity and stability.

**Figure 1:**
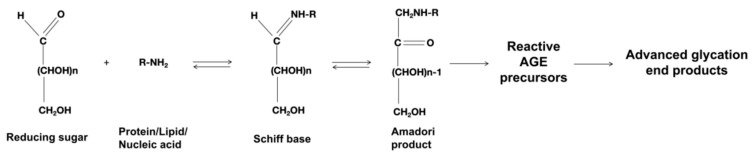
Structure and formation of advanced glycation end products. The Maillard reaction starts from the reaction of the aldehyde group in reducing sugar with the amino group in proteins and lipids, releases water molecules, and produces the Schiff base, which is reversible. After that, the product can be hydrolyzed again or further rearranged into a more stable Amadori product. Amadori products may undergo further dehydration and rearrangement to form AGEs, which is irreversible.

The Maillard reaction, shown in Figure 1, starts with interactions between the aldehyde groups found in reducing sugars (such as glucose, fructose, glyceraldehyde, and carbonyl compounds, such as acetaldehyde) and the amino groups in proteins or nucleic acids or lipids [28]. This process releases water molecules and results in the formation of an imine intermediate, also known as a Schiff base, which is reversible. The Schiff base may undergo hydrolysis or rearrangement to generate more stable Amadori products [3,19,29]. These Amadori products then undergo complex rearrangements, oxidations, reductions, dehydration, and cyclizations, ultimately forming stable AGEs [30,31]. This procedure potentially encompasses both oxidative and non-oxidative routes, yet it should be noted that both of these pathways are irreversible [28]. In the polyol pathway, the end products and their intermediates are Heyns products generated by the Heyns rearrangement. These then undergo a series of complex rearrangements, ultimately resulting in the formation of AGEs and a variety of heterogeneous reactive carbonyl compounds [14].

The chemical structure of these end products is diverse and includes a variety of distinct molecules with different properties [14]. The most well-known and widely studied AGE is CML, which is formed when the lysine residue of a protein reacts with glucose [32,33]. CML is a key marker of AGE accumulation and has been implicated in various diseases, including diabetic nephropathy [34]. Other important AGEs include pentosidine, a cross-linking product formed between pentose sugars and proteins, which has been shown to affect collagen and elastin in tissues, contributing to the stiffening of the extracellular matrix [35]. Additionally, pyrraline and glycated albumin are examples of other AGEs, each with distinct characteristics and tissue-specific effects [35].

The structure of CML consists of a stable heterocyclic ring formed by the reaction of reducing sugars with the amino group of lysine, and this structure is resistant to hydrolysis. CML can cross-link proteins, altering their structural integrity and function. The formation of cross-links between proteins, particularly in extracellular matrix components like collagen, can increase tissue stiffness and contribute to the aging process and various chronic diseases [33]. On the other hand, pentosidine, another commonly studied AGE, is formed by the reaction of reducing sugars with arginine residues. It forms a cross-linked structure, and its accumulation in tissues is associated with impaired protein function and the development of fibrosis, especially in the kidneys and the cardiovascular system [36].

The diversity of AGEs’ chemical structures leads to a wide range of biological activities. While some AGEs form through simple glycation, others involve the interaction of reducing sugars with proteins to generate more complex cross-links [14]. These AGEs accumulate in long-lived tissues, including the kidney, where they play a significant role in the pathophysiology of CKD [5]. AGEs can induce oxidative stress, activate inflammatory pathways, and promote fibrosis through their interaction with the receptor for advanced glycation end products (RAGE), a multiligand receptor that mediates many of the adverse effects of AGEs [37]. The molecular structure of AGEs thus affects not only their ability to cross-link proteins but also their potential to trigger cellular responses that drive disease progression.

## 4. AGEs and Kidney Diseases

### 4.1. Diabetic Nephropathy

As the most common complication in diabetic patients and the major cause of end-stage kidney disease (ESKD) worldwide, diabetic nephropathy is a chronic kidney disease characterized by a progressive decrease in the estimated glomerular filtration rate (eGFR) and proteinuria caused by a prolonged history of diabetes [38,39]. Every year, approximately 40% of patients with type 2 diabetes (T2DM) will develop DN, which accounts for 30% to 50% of ESKD, representing a major cause of death and disability among diabetic patients [40]. The risk of DN is linked to chronic hyperglycemia [41]. Despite the success achieved by strict blood glucose management and other therapeutic methods in preventing DN, they still fall far from achieving the desired results, and the tally of patients afflicted with end-stage diabetic kidney disease keeps climbing [5,42]. Additionally, in patients with chronic T2DM, the management of blood glucose levels appears to have a comparatively minor role in slowing down the progression of DN [43,44]. This implies that, apart from blood glucose, other factors linked to diabetes may play an important role in the deterioration of kidney function. In a hyperglycemic environment, the endogenous formation of AGEs increases [45,46]. A growing number of studies have revealed that AGEs are closely associated with the onset and progression of diabetic nephropathy. Table 1 summarizes the evidence on the associations of AGEs with diabetic nephropathy [10,38,47,48,49,50,51,52,53,54,55]. The kidney stands as the primary organ where AGEs accumulate in T2DM. The buildup of AGEs in the body can cause structural damage and functional loss in the kidney, ultimately leading to the progression of DN [56,57].

In a prospective cohort study utilizing the Hong Kong Diabetes Biobank, skin autofluorescence (SAF) was found to be closely associated with kidney disease in patients suffering from T2DM [49]. Similarly, two additional cohort studies based in the United States, namely the Action to Control Cardiovascular Risk in Diabetes (ACCORD) study (*n* = 1150 participants) and the Veterans Affairs Diabetes Trial (VADT) study (*n* = 447 participants), also demonstrated a strong correlation. In these two distinct T2DM cohorts, AGEs were significantly linked to a reduced eGFR and adverse renal outcomes, such as massive albuminuria [51]. Studies by Genuth S. et al. and Beisswenger, P. J. et al. revealed that serum and tissue AGE levels correlate with proteinuria in patients with type 1 diabetes (T1DM) [58] and with the progression of nephropathy in those with T2DM [59]. Compared to diabetic patients without kidney disease, those with ESKD exhibit approximately twice the amount of tissue AGEs [48]. Additionally, elevated circulating concentrations of AGEs and the soluble receptor for AGEs (sRAGE) positively correlate with the morbidity [50] and mortality [54] rates of DN. Ding. et al. discovered that the concurrent detection of AGEs enhances the reliability of an early diagnosis for DN [38]. The AGE-RAGE interaction assumes a pivotal role in the progression of DN. Yamamoto. et al. discovered that renal damage accelerates in diabetic mice with RAGE overexpression [60]. Furthermore, some clinical trials have demonstrated that drugs targeting AGE inhibition have a protective effect on the kidneys of patients with DN, but these trials are still in the first stage [61]. Research indicates that AGEs are linked to the severity of DN lesions in diabetic patients, hinting that they might serve as a crucial target for the prevention and management of DN.

### 4.2. Chronic Kidney Disease

Chronic kidney disease is defined as a condition characterized by persistent abnormalities in urine, kidney structure, or kidney function (a history of kidney damage of greater than 3 months), resulting in the loss of functional nephrons [62]. Estimates suggest that the global prevalence of chronic kidney disease stands at 14%, leading to 1.43 million deaths worldwide [62]. The relationship between elevated AGE levels and CKD has been documented for some time [46,63,64]. Table 2 summarizes the population-based studies on the role of AGEs in CKD [65,66,67,68,69,70,71,72]. In 2009, a piece of research concentrating on elderly female CKD patients demonstrated that elevated serum CML and sRAGE levels correlate with a decreased eGFR [73]. Another investigation (*n* = 1874) analogously discovered that variables related to kidney function were associated with elevated levels of AGE-CML, sRAGE, and esRAGE, showing a stronger correlation than diabetes-related indicators [67]. In an extensive cross-sectional study, increased levels of serum CML were linked to CKD and exhibited a negative correlation with eGFR [74]. In addition, the level of circulating AGEs increases with the progression of CKD [75], potentially rising 5–100 times in patients with ESKD [76]. AGE-free adducts serve as the principal means through which AGEs are removed from the body’s system. In patients with CKD, the impaired clearance mechanism significantly impacts the plasma levels of AGE-free adducts [77,78]. As dialysis cannot effectively eliminate AGEs [79], in comparison with healthy individuals, those undergoing hemodialysis (HD) or peritoneal dialysis (PD) display higher levels of circulating AGEs [80,81]. In patients with uremia, circulating AGEs show a positive correlation with indicators of inflammation and oxidative stress [82] and predict CVD mortality among stable renal transplant recipients [70]. SAF serves as an indicator for measuring the accumulation of AGEs. A multi-institutional prospective cohort study involving 1634 HD patients revealed that baseline SAF is significantly linked to the risk of all-cause and CVD mortality [69]. Furthermore, in CKD patients, regardless of their diabetes status, SAF increases significantly [83,84,85], negatively correlates with eGFR [86,87], positively correlates with mortality [88], and predicts the progression of CKD [83,84]. These findings indicate that AGEs are not merely biomarkers of glucose homeostasis; their production and accumulation are closely linked to CKD.

In addition, AGEs derived from the diet also have a substantial influence on the initiation and development of CKD. Several animal-based research efforts have illustrated a connection between AGEs derived from the diet and renal impairment: Wistar rats, previously without any renal history, exhibited proteinuria after being fed a high-AGE diet (the high-AGE diet is an AIN-93G diet exposed to heat, promoting the formation of AGEs) for six consecutive weeks [89]. In contrast to mice maintained on a low-AGE diet, non-obese diabetic mice on a long-term high-AGE diet showed higher AGE levels in their serum and kidneys, along with more severe glomerular lesions and increased urinary albumin [90]. Among 5/6 nephrectomized rats, those on a low-AGE diet demonstrated reduced proteinuria and a slower decline in the creatinine clearance rate compared to rats fed either a standard or a high-AGE diet (5–13 weeks) [91]. In population trials, AGE-restricted diets were linked to a decrease in oxidative stress markers and circulating AGEs, yet the impact of such diets on proteinuria and/or the real-world function of the kidneys was still unsubstantiated [71,92]. Among those undergoing peritoneal dialysis, it has been revealed that a low-AGE diet is capable of reducing the levels of AGEs in circulation. Specifically, a study involving non-diabetic ESKD patients undergoing sustaining peritoneal dialysis randomly assigned them to either a conventional or a low-AGE diet. Notably, the low-AGE diet group exhibited an important reduction in circulating AGEs and C-reactive protein levels [93]. Another parallel intervention among peritoneal dialysis patients further confirmed the effectiveness of a low-AGE diet in lowering circulating AGE levels [94]. Several other clinical studies that have been conducted aimed at reducing the dietary exposure to AGEs in patients with CKD through intervention measures to improve vascular health [92,95]. These studies typically also evaluated the markers of vascular inflammation. The findings imply that diminishing the content of AGEs in regular diets could potentially decrease oxidative stress and inflammation. Moreover, it may restore the levels of AGE receptor 1 (AGER1), which functions as an antioxidant, in healthy individuals, the elderly, and patients with CKD-3 [92]. In addition, overweight and obese participants were enlisted in a randomized, crossover clinical trial that consisted of two-week intervals on a low-AGE diet and a high-AGE diet. Among the obese subjects, urinary albumin to creatinine ratios (ACR) notably improved following the low-AGE diet period [95]. A significant statistical difference was found when comparing the low-AGE diet with the high-AGE diet (*p* = 0.02) [95]. There is only a limited amount of research regarding the long-term impacts of a diet rich in AGEs on the risk of kidney disease among healthy individuals. A prospective study utilizing a dietary questionnaire indicated that consuming significant amounts of AGEs from dietary fat sources could double the risk of CKD [72]. However, there is an absence of population-wide trials that investigate the direct effects of AGEs from the diet on the functionality of the kidneys.

### 4.3. Acute Kidney Injury

Acute kidney injury is a rapid decline in kidney excretory function, which can elevate the incidence of ESKD by 13 times [96]. In critically ill patients, the prognosis is often poor and the mortality is very high [96]. A study revealed that AKI was more severe in mice deficient in both the RAGE and the sRAGE compared to wild-type (WT) controls, showing increased renal tubular damage, macrophage infiltration, and fibrosis [97]. In terms of population studies, a cross-sectional study involving 40 patients with AKI and 39 age-matched, healthy controls found that RAGE-binding protein was elevated in the AKI patients compared with the control group [98]. Similarly, another study conducted on post-traumatic patients also found that the RAGE was significantly elevated in patients with AKI at most time points in comparison to the control group [99]. In addition, in critically ill patients with AKI, higher sRAGE concentrations are frequently detected, correlating with organ failure and mortality [100,101]. In a prospective study targeting patients with sepsis-induced AKI, RAGE levels demonstrated a positive correlation with E-selectin (r = 0.88), endothelin-1 (ET-1) (r = 0.90), and tumor necrosis factor-α (TNF-α) (r = 0.94) levels, while being inversely correlated with NO levels [102]. These findings imply that circulating AGEs could be linked to the disease’s severity and outcomes. Nevertheless, a separate small study observed no increase in blood RAGE levels during AKI [98]. Given that there are relatively few studies available at present, further high-quality research is required to investigate the relationship between AGEs and AKI.

### 4.4. Other Kidney Diseases

Studies have shown that AGEs are involved in the development of various other kidney diseases: AGE staining is observed in the glomeruli of focal segmental glomerulosclerosis, hypertensive glomerulosclerosis, and lupus nephritis, with the deeper staining of AGEs seen in damaged glomeruli (such as sclerosing glomeruli, nodular lesions, and ischemic crescent formations) [103,104]. A cross-sectional study involving 28 patients with familial amyloidotic polyneuropathy (FAP) and 18 healthy controls revealed the buildup of AGEs, the RAGE, and amyloid in the kidneys of FAP patients [105]. However, the direct role of AGEs in the development of these kidney diseases has yet to be clarified.

## 5. Possible Mechanisms of AGEs on Kidney Damage

**Figure 2:**
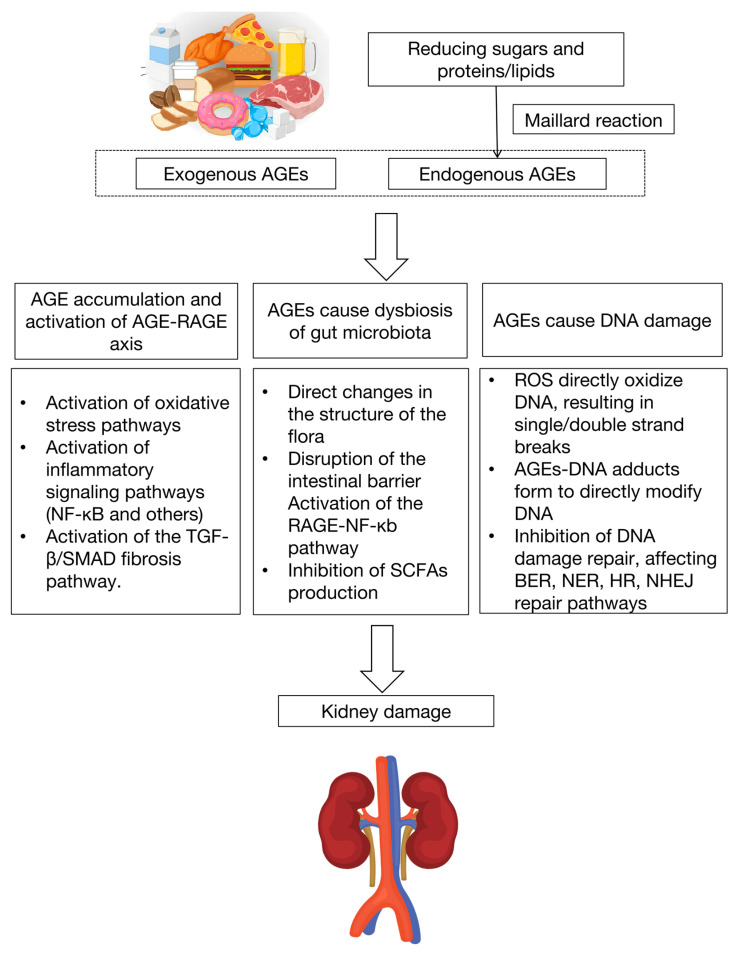
Systemic AGEs can be formed either endogenously or exogenously in patients with kidney diseases. AGEs accumulate in the body and cause kidney dysfunction through multiple pathways (AGE-RAGE axis, gut microbiota, DNA damage). AGEs, advanced glycation end products; RAGE, receptor for AGEs; ROS, reactive oxygen species; TGF-β, transforming growth factor-β; SMAD, Suppressor of Mothers Against Decapentaplegic; SCFAs, short-chain fatty acids; BER, base excision repair; NER, nucleotide excision repair; HR, Homologous Recombination Repair; NHEJ, Non-Homologous End Joining.

The possible mechanistic pathways for AGEs’ contribution to kidney damage are summarized in Figure 2.

### 5.1. Accumulation of AGEs and AGE-RAGE Axis

The kidney, as the primary structure for the metabolism and excretion of AGEs, is one of the main sites of AGE accumulation. Low-molecular-weight AGEs are capable of being freely filtered through the glomerulus, while high-molecular-weight AGEs undergo lysosomal degradation or autophagy within the renal tubules [24]. In the kidneys, AGEs are primarily formed through the interaction of glucose and other reducing sugars with proteins such as collagen and albumin, leading to the formation of various AGE compounds, including CML and pentosidine [106]. These AGEs accumulate in the glomerular basement membrane, tubules, and interstitial spaces, impairing the structural integrity and function of the kidney [2,107]. The elevated levels of AGEs in renal tissues have been linked to increased fibrosis, glomerulosclerosis, and tubulointerstitial damage, which are hallmark features of CKD progression [63,108]. Studies have shown that AGE accumulation in the renal vasculature, particularly in the glomeruli and proximal tubules, is associated with increased oxidative stress and inflammation, which can lead to endothelial dysfunction and renal fibrosis [2,6,108].

The excessive accumulation of AGEs can impair the protein exocytosis of renal tubular cells. Furthermore, AGEs induce protein function and tissue structure damage through in situ glycosylation, such as (1) the cross-linking of matrix proteins, which results in increased rigidity and alters the structure and function of the glomerulus and the vascular system surrounding the renal tubules and arterioles, contributing to glomerulosclerosis, atherosclerosis, and the thickening of the basement membrane [109]; (2) the accumulation of AGEs in the glomerulus, which induces podocyte dedifferentiation, further intensifying the inflammatory response [28]; and (3) AGE accumulation, which activates ROS and cell apoptosis. These factors collectively contribute to or worsen hemodynamic alterations, glomerulosclerosis, tubulointerstitial fibrosis, proteinuria, albuminuria, and a decline in the glomerular filtration rate. As renal function deteriorates, the excretion of AGEs by the kidneys diminishes, resulting in their increased buildup in the body. This establishes a self-perpetuating cycle that further aggravates kidney injury [46,110,111,112,113,114].

AGEs exert their detrimental effects on renal tissues primarily through the activation of the receptor for the RAGE, which is expressed on a variety of renal cell types, including mesangial cells, podocytes, and endothelial cells [6,57]. High AGE concentrations in vitro trigger RAGE-dependent epithelial-to-myofibroblast transformation, leading to tubulointerstitial fibrosis [113,115,116]. The interaction between AGEs and the RAGE triggers the activation of key signaling pathways, including nuclear factor kappa B (NF-κB) and Janus kinase—a signal transducer and the activator of transcription (JAK-STAT) [117,118,119,120,121,122]. This activation ultimately induces the production of various pro-inflammatory cytokines and chemoattractants [117,118,119,120,121,122]. This promotes inflammation via the interleukin-1β and TNF-α pathways [123,124], as well as stimulates the production of ROS originating from nicotinamide adenine dinucleotide phosphate (NADPH) oxidase and mitochondrial activity [125,126]. Through these pathways, AGEs exacerbate renal fibrosis, glomerulosclerosis, apoptosis, and cell death, ultimately causing kidney damage and a decline in kidney function. The accumulation of RAGE ligands, such as AGEs, leads to an upregulation of RAGE expression on podocytes in both human and rodent models [104,126,127]. Numerous studies have demonstrated that compared to aged wild-type mice, elderly RAGE gene knockout (KO) mice exhibit reduced levels of pro-inflammatory cytokines and attenuated glomerulosclerosis, a marker of kidney aging [125,128,129,130].

Therefore, the accumulation of AGEs in kidney tissues plays a significant role in the pathogenesis of diabetic nephropathy and other kidney diseases, making AGE levels a potential biomarker for disease progression and a target for therapeutic intervention.

### 5.2. AGEs Cause Dysbiosis of Gut Microbiota

A diet rich in AGEs can influence the composition and function of the gut microbiota [131]. Recent research indicates that gut microbiota dysbiosis is also crucial in accelerating kidney damage [132,133]. Apart from being produced endogenously in the body, AGEs are also significantly obtained through dietary intake. The intake of dietary AGEs positively correlates with serum AGE levels [134]. Furthermore, dietary AGEs may accumulate in gastrointestinal tissues, leading to the disruption of microbial communities. Studies by Wu [135] and Seiquer [136] demonstrate that a high dietary intake of AGEs has a pronounced impact on the gut microbiota, characterized by an increased population of Bacteroidetes, a decreased abundance of Firmicutes, and a reduced relative abundance of lactic acid bacteria. Intestinal dysbiosis, barrier dysfunction, and bacterial translocation contribute to systemic inflammation in CKD [137], which may aggravate endothelial dysfunction, arteriosclerosis, and CVD [138]. In addition, a diet high in AGEs can also lower the levels of glycomic bacteria, which are associated with the production of short-chain fatty acids (SCFAs) [139]. Recent research suggests that the SCFAs produced by the gut microbiota influence the severity of AKI by regulating immune and inflammatory responses [140]. Mishima et al. indicated that in germ-free mice, the gut’s SCFA production is notably reduced, potentially exacerbating adenine-induced kidney damage [141]. This implies that exposure to intestinal AGEs can decrease SCFA production, thereby elevating the risk of kidney injury progression. Furthermore, studies have revealed that high dietary exposure to AGEs can downregulate tight junction proteins in epithelial cells [133]. An elevated dietary AGE intake can impact the tight junctions of intestinal epithelial cells, compromise the intestinal epithelial barrier, and trigger “intestinal leakage” in db/db mice, potentially worsening kidney damage [142]. Therefore, reducing dietary AGE intake or therapeutically removing AGEs from the gastrointestinal tract could serve as a promising strategy to preserve the gut microbiota balance and mitigate the progression of kidney damage.

### 5.3. AGE Influence on DNA Damage Formation and Its Repair

An emerging area of research is the role of AGEs in DNA damage and the subsequent repair mechanisms in renal cells. AGEs induce oxidative DNA damage, primarily through the generation of reactive oxygen species (ROS) upon their interaction with the RAGE [143,144]. ROS generated in response to AGEs can cause DNA strand breaks, base modifications, and the cross-linking of DNA molecules, leading to genomic instability [144,145,146]. This type of damage is particularly concerning in kidney disease, where prolonged exposure to AGEs can impair renal cell function and survival.

Furthermore, AGEs interfere with DNA repair mechanisms, particularly in kidney tissues. DNA repair processes, such as base excision repair (BER) and nucleotide excision repair (NER), are essential for maintaining genomic integrity [147,148]. However, the accumulation of AGEs appears to impair these processes, hindering the repair of DNA damage. This leads to genomic instability, increasing the likelihood of mutations and fibrosis [148]. The impairment of DNA repair pathways may also result in cellular senescence and apoptosis, both of which contribute to kidney dysfunction and fibrosis [149,150].

The inability to repair AGE-induced DNA damage exacerbates renal dysfunction and accelerates the progression of kidney diseases [151]. Studies have shown that AGE-induced DNA damage impairs DNA repair activity in renal cells, which can lead to reduced cell viability, genomic instability, and fibrosis in kidney tissues [146,147]. This suggests that enhancing DNA repair mechanisms and targeting AGE-induced damage may offer novel therapeutic strategies to prevent or slow the progression of kidney diseases.

## 6. AGEs in Early Diagnosis, Treatment, and Prognosis of Kidney Disease

### 6.1. Early Diagnosis

AGE accumulation is a hallmark of kidney disease, especially in DN and CKD, where it directly contributes to the pathological changes that lead to renal dysfunction [28,152]. Earlier studies have suggested that AGEs specifically bind to the Endothelium and affect its function, leading to abnormal coagulation and increased permeability, which may play a key role in diabetes and aging-related vasculopathy [152]. AGEs interact with specific receptors, such as the RAGE, which triggers inflammatory responses, oxidative stress, and apoptosis in kidney cells, accelerating kidney damage [5,153,154]. Due to their ability to accumulate in tissues before significant changes in traditional renal markers like serum creatinine and albuminuria, AGEs can serve as early biomarkers for kidney disease [16,155]. These early biomarker capabilities have been highlighted in several studies, with evidence suggesting that AGE levels may be elevated even in the initial stages of kidney dysfunction, preceding changes in conventional markers of kidney injury [155,156].

Recent studies have shown that AGE markers, CML and pentosidine, are elevated in patients with CKD and diabetic nephropathy, correlating with early kidney dysfunction [10,38,156]. Additionally, AGE accumulation in urine has been associated with decreased eGFR, and the detection of AGE levels in both serum and urine can provide insights into early kidney damage [38,157,158]. Monitoring AGEs offers the advantage of detecting kidney dysfunction before traditional markers become abnormal, enabling earlier diagnoses and intervention.

Current methods for measuring AGEs include the enzyme-linked immunosorbent assay (ELISA), mass spectrometry, and immunohistochemical staining [159]. These techniques have demonstrated high sensitivity and specificity, making AGE measurement a reliable tool for detecting kidney damage. As such, AGE testing can complement traditional renal function tests, providing a more comprehensive approach to early diagnosis and management.

### 6.2. Treatment

Given their significant role in kidney disease progression, targeting AGEs has become an attractive therapeutic strategy for managing diabetic nephropathy and CKD. Several pharmacological approaches aim to either inhibit AGE formation or break AGE-protein cross-links to reduce their harmful effects [57].

One class of drugs under investigation are AGE inhibitors, which block the formation of AGEs from reducing sugars. These include compounds such as aminoguanidine, benfotiamine, and pyridoxamine, which have shown some success in animal models and clinical trials [57]. Aminoguanidine, for example, has been shown to reduce AGE formation and improve renal function in diabetic nephropathy [160]. However, its clinical application has been limited due to side effects and toxicity at higher doses. Benfotiamine, a synthetic form of vitamin B1, has demonstrated promising results in reducing AGE formation by enhancing the activity of transketolase, an enzyme involved in the pentose phosphate pathway, which helps to counteract sugar toxicity and AGE formation [161,162]. Moreover, pyridoxamine (a vitamin B6 derivative) has been shown to prevent the formation of AGEs and improve renal function in diabetes-induced kidney damage [163]. Another approach is targeting the AGE-RAGE interaction itself. Soluble RAGE, a decoy receptor that can bind AGEs and prevent them from interacting with cell surface RAGE, has been proposed as a potential therapeutic strategy [6,164]. Studies have shown that soluble RAGE can reduce AGE-induced inflammation and fibrosis in kidney tissues, thereby protecting against renal injury [57]. In addition, the small-molecule inhibitors of the RAGE are being actively investigated. These inhibitors, such as RAGE antagonists or small peptides that block RAGE binding, have shown potential in reducing AGE-induced inflammation and tissue damage in animal models of diabetes and chronic kidney disease [165,166].

In addition, another promising strategy involves the use of AGE cross-link breakers, like ALT-711. This compound has been demonstrated to improve kidney function and reduce fibrosis in experimental models of diabetic nephropathy [167]. ALT-711 works by disrupting the cross-links formed between AGEs and proteins, which are responsible for the stiffness and fibrosis seen in damaged tissues [168]. Additionally, lifestyle interventions, such as dietary modifications aimed at reducing AGE intake, have shown beneficial effects in reducing AGE accumulation and slowing the progression of kidney disease. Studies have found that limiting the consumption of foods rich in AGEs, such as those cooked at high temperatures, can significantly decrease AGE levels in the body, potentially improving kidney function [1,12].

While these treatments show promise, further well-designed, randomized, controlled trials are necessary to confirm their efficacy in human populations. Future strategies may involve a combination of pharmacological treatments and dietary modifications tailored to individual patients, offering a comprehensive approach to managing kidney disease progression.

### 6.3. Prognosis

In addition to serving as biomarkers for early diagnoses, AGEs are also valuable indicators of disease progression and prognoses in kidney diseases. Studies have demonstrated that elevated AGE levels are associated with the severity of kidney damage and the risk of progression to ESKD [161]. In CKD patients, high AGE levels correlate with increased fibrosis, glomerulosclerosis, and tubulointerstitial injury, all of which accelerate kidney dysfunction [5,169]. Furthermore, AGEs are strongly associated with cardiovascular complications, which are a leading cause of morbidity and mortality in CKD patients [161,170].

The accumulation of AGEs in kidney tissues contributes to the development of fibrosis by activating fibroblasts and increasing extracellular matrix production, ultimately leading to kidney scarring and the loss of function [171]. AGEs also impair vascular function by promoting endothelial dysfunction and increasing arterial stiffness, which is commonly observed in CKD patients [161,172]. Therefore, measuring AGE levels can help predict not only kidney disease progression but also the risk of cardiovascular events and overall mortality.

Recent studies have emphasized the prognostic value of AGE measurements in CKD patients. Elevated serum and urinary AGE levels have been associated with poor renal outcomes and a higher risk of ESRD and cardiovascular events [161,173]. Monitoring AGE levels can help clinicians assess the risk of disease progression and tailor treatment plans more effectively, potentially improving the long-term outcomes for CKD patients [5].

## 7. Conclusions

Since Maillard first discovered the Maillard reaction in 1912 [174], research into AGEs has never ceased. Dietary AGEs are widely prevalent in contemporary diets, and the overall increase in both endogenous and exogenous AGEs is implicated in the pathogenesis of numerous diseases. In this review, we presented evidence elucidating the impact of AGEs on kidney function and its potential mechanisms. AGEs can impair kidney function and tissue structures through accumulation in renal tissue and also activate downstream signaling pathways via the AGE-RAGE axis, triggering oxidative stress, inflammation, and other processes, ultimately exacerbating renal damage. Furthermore, intestinal flora imbalance significantly contributes to kidney injury induced by AGEs. These findings suggest that AGEs may serve as a key driver in the progression of kidney diseases and could potentially be developed as a novel marker for kidney dysfunction, significantly aiding in the diagnosis of DKD, the prognosis prediction of CKD, and disease surveillance. However, it remains unclear whether the functional and structural alterations in the kidney are directly attributed to the kidney’s uptake and transport of AGEs or if they are indirectly mediated by AGEs’ effects on blood vessels, the gastrointestinal tract, the intestinal endocrine system, and the microbiome, with subsequent renal interactions. Additionally, considering the widespread presence of AGEs in modern diets, reducing their intake may contribute to enhancing kidney health. Consequently, further in-depth research is warranted to elucidate the role of AGEs in kidney diseases and kidney function, aiming to offer novel preventive and therapeutic approaches to improve public health.

## Figures and Tables

**Table 1:** Summary of evidence on the associations of AGEs with diabetic nephropathy.

Author (Year)	Study Type	Country	Population	Detection Method	Outcome/Purpose	Results
Ding, L. et al., 2024 [38]	Case-control	China	T2DM:176 (Divided into three groups according to UACR); Control: 34	AGEs, CML, MGO: ELISA	Kidney function (U-Alb, UACR, and blood urea nitrogen).	Higher serum AGE levels were found to be positively correlated with U-Alb, UACR, and blood urea nitrogen in the study of 176 individuals with type 2 diabetes. CML and MGO levels were positively correlated with U-Alb, UACR, blood urea nitrogen, Scr, and uric acid, and negatively correlated with estimated glomerular filtration rate (*p* < 0.05). A multivariate logistic regression analysis showed that elevated levels of AGEs, CML, and MGO were independent risk factors for the progression of DN (OR = 1.861, 1.016, 7.607, *p* < 0.01).
Monnier, V.M. et al., 2022 [52]	Cohort	USA, Canada	DCCT/EDIC 466 T2DM	AGEs: LC-MS	To assess impact of glycemic control on pAGEs and their association with subsequent microvascular disease.	MetSOX is linked to DN at TP1 site. When age, the BMI, the duration of DM, and the mean updated HbA1c were considered, there was a positive correlation between fructose lysine and CML and albuminuria.
Koska, J. et al., 2022 [51]	Cohort	USA	ACCORD:1150 T2DM VADT:447 T2DM	AGEs: LC-MS	Kidney function (eGFR, macroalbuminuria) RFL, hrCKD.	AGE score was associated with reduction in eGFR (β-estimate −0.66 mL/min /1.73 m^2^ per year; *p* = 0.001), 30% RFL (HR 1.42 [95% CI 1.13–1.78]; *p* = 0.003), 40% RFL (1.40 [1.13–1.74]; *p* = 0.003), macroalbuminuria (1.53 [1.13–2.06]; *p* = 0.006), and hrCKD (1.88 [1.37–2.57]; *p* < 0.0001).
Jin, Q et al., 2022 [49]	Cohort	China HK	HKDB: 3725 T2DM	SAF	Kidney dysfunction.	SAF was associated with progression of kidney disease (HR 1.15 per SD, 95% CI [1.04, 1.28]) and annual decline in eGFR (β −0.39 per SD, 95% CI [−0.71, −0.07]) after adjustment for risk factors, including baseline eGFR and UACR. Decreased eGFR (12.9%) and increased UACR (25.8%) accounted for 38.7% of the effect of SAF on renal outcome.
Steenbeke, M. et al., 2022 [53]	RCT	The Netherlands	T2DM DN low-AGE (*n* = 20); regular diabetic diet (*n* = 20)	AGEs: SAF and Urine spectrometry; sRAGE: ELISA	Skin and urine, or sRAGE.	An AGE-limited diet for two months did not affect the AGE content in skin and urine, nor the sRAGE concentration in the blood.
Wong, F.N. et al., 2018 [55]	Case-control	Malaysia	DN (*n* = 150) non-DN (*n* = 64)	sRAGE: ELISA	To explore the effect of blood glucose control on the level or activity of sRAGE in patients with CKD.	Glycemic control did not quantitatively alter GPx, SOD, or sRAGE in diabetic CKD patients.
Dozio, E. et al., 2017 [48]	Cross-sectional	Italy	DM CKD-G5D (*n* = 24) non-DM CKD-G5D (*n* = 52)	sRAGE: ELISA	Assessing the association between AGEs and an increased CV risk in CKD-G5D patients.	Compared with non-DM CKD-G5D patients, DM CKD-G5D patients have higher sRAGE levels.
Klein, R. et al., 2017 [50]	Cohort	USA	697 T1DM	sRAGE and CML: ELISA	To study the relationship between the levels of CML and sRAGE and the risk of DKD.	Higher levels of sRAGE were significantly associated with the incidence of DN ([HR] 1.12 per 0.2 log pg/mL [95% CI 1.04, 1.20]).
Saulnier, P.J. et al., 2016 [10]	Cohort	USA	169 T2DM	AGEs: LC-MS	RFL.	Each doubling of CML ([HR] 1.60 [95% CI 1.08–2.37]) or MGHI (HR 1.30 [95% CI 1.02–1.65]) concentration was associated with RFL.
Thomas MC et al., 2015 [54]	Case-control	Asia, Australasia, Europe, and North America	3763 T2DM	sRAGE: ELISA	New or worsening nephropathy.	sRAGE levels were associated with new or worsening nephropathy (HR 1.20 for a 1-SD increase of log sRAGE [95% CI 1.02–1.41]; *p* = 0.032). Circulating AGE levels were also independently associated with new or worsening nephropathy (HR 1.21 for a 1-SD increase [95% CI 1.08–1.36]; *p* = 0.001).
Beisswenger, P.J. et al., 2013 [47]	Cohort	NA	103 T1DM	AGEs: LC/MS-MS	Change in GBM width from baseline to 5 years, measured using electron micrographs of renal biopsies, was our primary end point, and mesangial fractional volume was a secondary end point. FPs were defined as those in the upper quartile of GBM change, and the remaining patients were classified as SPs.	MGHI, CEL, and CML levels were significantly higher in FPs relative to SPs.

Abbreviation: T2DM: type 2 diabetes; UACR: urinary creatinine to albumin ratio; AGEs: advanced glycation end products; CML: N6-carboxymethyl-l-lysine; MGO: methylglyoxal; ELISA: enzyme-linked immunosorbent assay; U-Alb: urinary albumin; Scr: serum creatinine; OR: odds ratio; DN: diabetic nephropathy; DCCT: the diabetes control and complications trial; EDIC: Epidemiology of Diabetes Interventions and Complications; LC: liquid chromatography; MS: mass spectrometry; pAGEs: plasma protein-bound advanced glycation end products; MetSOX: methionine sulfoxide; TP: timepoint; BMI: body mass index; DM: diabetes mellitus; HbA1c: hemoglobin A1c; ACCORD: Action to Control Cardiovascular Risk in Diabetes; VADT: Veterans Affairs Diabetes Trial; eGFR: estimated glomerular filtration rate; RFL: renal function loss; hrCKD: high-risk chronic kidney; HR: hazard ratio; CI: confidence interval; HKDB: Hong Kong Diabetes Biobank; SAF: skin autofluorescence; SD: standard deviation; RAGE: receptor for AGEs; sRAGE: soluble RAGE; RCT: randomized controlled trial; CKD: chronic kidney disease; GPx: glutathione peroxidase; SOD: superoxide dismutase; CKD-G5D: CKD stage 5 on dialysis; T1DM: type 1 diabetes; MS-MS: tandem MS; CEL: N6-carboxyethyl-l-lysine; MGHI: methylglyoxal hydroimidazolones; FPs: Fast progressors; SPs: slow progressors; GBM: glomerular basement membrane.

**Table 2:** Summary of evidence on the associations of AGEs with CKD.

Author (Year)	study Type	Country	Population	Detection Method	Outcome/Purpose	Results
Molinari P. et al., 2021 [65]	Cohort	USA	CKD (*n* = 65)	AGEs: fluorescence spectrophotometry. sRAGE: ELISA.	Evaluate the changes of AGEs and sRAGE in patients with chronic kidney disease at different time points.	AGEs and the AGEs/sRAGE ratio were negatively correlated with eGFR, but changes in AGE and RAGE subtypes were not related to changes in eGFR.
Dozio E. et al., 2020 [66]	Cohort	Italy	CKD 3b-5 (*n* = 111)	AGEs: fluorescence spectrophotometry. sRAGE: ELISA.	we explored the role of AGEs, glycated albumin, and sRAGE and its different forms, cRAGE and esRAGE, as prognostic factors for mortality in advanced CKD patients.	After 39 months of follow-up, eGFR showed a negative correlation with AGE, sRAGE, esRAGE, and cRAGE.
Loomis S.J. et al., 2017 [67]	Cross-sectional	USA	ARIC participants (*n* = 1874)	AGEs (CML): ELISA. sRAGE: ELISA.	To assess the association of AGE-CML, sRAGE, and esRAGE with indicators of diabetes and kidney disease.	The risk of albuminuria and CKD is associated with elevated levels of AGE-CML, sRAGE, and esRAGE.
Prasad K. et al., 2016 [68]	Case-control	Canada	ESKD (*n* = 88); health control (*n* = 20)	AGEs: ELISA. sRAGE: ELISA.	Study the correlation between AGE levels and sRAGE, as well as the increase in the AGEs/sRAGE ratio in ESKD patients.	The levels of AGEs in ESKD patients were 6.75 times higher than those in the control group. The increase in AGE levels is 2–3.23 times that of sRAGE levels. There is a negative correlation between sRAGE and the AGEs/sRAGE ratio.
Jiang J. et al., 2021 [69]	Cohort	China	CCSD HD patients (*n* = 1634)	AGEs: fluorescence spectrophotometry, SFA.	Study the association between serum and tissue AGEs and all-cause and CVD mortality rates.	Baseline tissue AGE levels are positively correlated with all-cause mortality and CVD mortality, but not with circulating AGEs or other important confounding factors.
Sotomayor, C.G. et al., 2019 [70]	Cohort	The Netherlands	Kidney transplant patients (*n* = 555)	AGEs: LC-MS.	The relationship between AGEs and cardiovascular mortality.	The concentrations of CML and CEL are directly correlated with cardiovascular mortality (HR: 1.55; 95% CI: 1.24–1.95; *p* < 0.001; HR: 1.53; 95% CI: 1.18–1.98; *p* = 0.002).
Baye, E et al., 2017 [71]	Randomized, double blind, crossover trial	Australia	over-weight (*n* = 20)	Alternate low-AGE and high-AGE diets for two weeks, with a clearance period of four weeks apart.	Can a low-AGE diet reduce inflammation and cardiovascular risk in overweight and obese healthy adults?	Inflammation markers (including AGEs, TNF α, vascular cell adhesion protein-1, and the RAGE) were significantly reduced in patients in the low-AGE diet group.
Ejtahed, H.S et al., 2016 [72]	Cohort	Iran	healthy adult (*n* = 3462)	Dietary AGEs: Semi Quantitative Food Frequency Questionnaire.	The association between dietary AGEs and CKD.	Higher consumption of AGEs through dietary fat was associated with higher risk of CKD incidence.

Abbreviation: CKD: chronic kidney disease; AGEs: advanced glycation end products; RAGE: receptor for AGEs; sRAGE: soluble RAGE; eGFR: estimated glomerular filtration rate; ELISA: enzyme-linked immunosorbent assay; cRAGE: cleaved RAGE; esRAGE: endogenous secretory RAGE; ARIC: Atherosclerosis Risk in Communities study cohort; CML: N6-carboxymethyl-l-lysine; DM: diabetes mellitus; ESKD: end-stage kidney disease; CCSD: the China Cooperative Study on Dialysis; HD: hemodialysis; SAF: skin autofluorescence; CVD: cardiovascular disease; LC: liquid chromatography; MS: mass spectrometry; HR: hazard ratio; CI: confidence interval; TNF: tumor necrosis factor.
